# Brief telephone counselling is effective for caregivers who do not experience any major life events – caregiver-related outcomes of the German day-care study

**DOI:** 10.1186/s12913-018-3853-8

**Published:** 2019-01-09

**Authors:** Elisa-Marie Behrndt, Melanie Straubmeier, Hildegard Seidl, Chiara Vetter, Katharina Luttenberger, Elmar Graessel

**Affiliations:** 1 Centre for Health Services Research in Medicine, Department of Psychiatry and Psychotherapy, Friedrich-Alexander-University Erlangen-Nürnberg (FAU), Schwabachanlage 6, 91054 Erlangen, Germany; 2 Institute of Health Economics and Health Care Management, Helmholtz Zentrum München, Ingolstädter Landstraße 1, 85764 Neuherberg, Germany

**Keywords:** Day-care, Dementia, MCI, Informal caregivers, Telephone intervention

## Abstract

**Background:**

To date, there has been a dearth of scientifically tested, established intervention concepts focussed on supporting informal caregivers and embedded in routine health care structures. The aim of this study was to assess effects of a brief telephone intervention for caregivers of persons with cognitive impairment (PCIs) on caregivers’ depressiveness and subjective burden.

**Methods:**

A two-arm cluster-randomised controlled intervention study was carried out at 32 German day-care centres. During the six-month intervention period, informal caregivers in the intervention group (*n* = 205) received counselling in three phone calls focussed on stress reduction, development of self-management strategies, and how to deal with challenging behaviours. Both the control group (*n* = 154) and the intervention group were free to take part in any support programmes offered by the German Health Care System (TAU). Caregivers’ subjective burden and depressiveness were measured with the Burden Scale for Family Caregivers – short version (BSFC-s) and the WHO-5 Well-Being Index (WHO-5). Outcomes were assessed by means of computer-assisted telephone interviews (CATIs) at baseline and at the end of the six-month intervention phase. Multiple regression analyses were used to show the influence of group allocation.

**Results:**

After the intervention phase, group allocation was not found to significantly predict caregivers’ subjective burden or depressiveness. The baseline scores (*p* < 0.001) were the only significant predictors of change in both outcomes. However, sensitivity analyses for caregivers who did not experience any events that they felt were major (in a negative or positive sense) during the six months (*n* = 271) showed that group allocation (*p* < 0.05) was a significant predictor of positive change in both outcomes (BSFC-s: Δ-1.3, [− 2.4, − 0.3], Cohen’s d = 0.27; WHO-5: Δ1.5, [0.4, 2.7], Cohen’s d = 0.26). Effect sizes were highest in the subgroup of caregivers of people with mild dementia (BSFC-s: Cohen’s d = 0.43; WHO-5: Cohen’s d = 0.42).

**Conclusions:**

A “low-dose” psychoeducative telephone intervention designed to empower caregivers is effective, especially in an early stage, if the overlap between the effect of the intervention and the effect of events that are experienced as major events in the caregiver’s life is considered.

**Trial registration:**

Identifier: ISRCTN16412551 (Registration date: 30 July 2014, registered retrospectively).

**Electronic supplementary material:**

The online version of this article (10.1186/s12913-018-3853-8) contains supplementary material, which is available to authorized users.

## Background

The deterioration of cognitive skills and the ability to carry out activities of daily living (ADL) in persons with cognitive impairment (PCIs) is often associated with a decline in independence. For informal caregivers, this can lead to an increase in caregiver burden, due to the necessity of assuming more and more tasks for the person in need of care [1]. In addition to the deficits in ADL and cognition, the “unexplainable” or challenging behaviours of people with dementia are particularly stressful for their caregivers [2].

At the same time, both parties often want the person with dementia to continue to live at home for as long as possible [3], which also has health economic advantages [4]. To achieve this goal, it is essential to begin when the disease is in its early stages. The preferred methods are supportive preventive arrangements that sustain the PCIs’ independence for as long as possible while also reducing the subjective burden experienced by informal caregivers.

The PCI-informal caregiver dyad can be contacted through an ambulatory care setting, especially day-care centres, since many of the users of such centres are elderly people with relatively pronounced cognitive deficits that range from mild cognitive impairment (MCI) to severe dementia [5]. Not only do the day-care centres provide care for the PCIs and increase their well-being [6], but they also relieve the burden on the users’ informal caregivers and give them support, e.g. by providing information about dementia-related topics [7]. In this context, a two-arm intervention study was developed [8].

In this study, in the treatment group, the informal caregivers of day-care centre users with cognitive impairment were supported by means of a low-threshold, outreach telephone counselling service, whereas caregivers in the control group received “care as usual,” which meant that the study centre did not intervene in any potential additional relief services that caregivers may have secured for themselves. Telephone counselling sessions for informal caregivers of people with dementia can improve caregivers’ emotional health [9]. However, these sessions cannot be assumed to be effective in all cases, as demonstrated by a large-scale German intervention study that failed to show significant differences between the groups with regard to subjective burden [10].

A literature search of the electronic databases PubMed, PsycINFO, Psyndex, Embase, and Cinahl for telephone-based caregiver interventions with a randomised controlled study design resulted in five hits. The interventions were described as either psychoeducative [11–14] or cognitive-behavioural [15]. All consisted of several components ranging from the management of behavioural and psychological symptoms of dementia [12] and stress management [13], as in our study, to directing caregivers to appropriate resources [14]. The intervention periods ranged from 3 to 12 months. In all cases, the frequency of the phone calls, i.e. the “treatment dose”, was greater than in our study. On average, the calls took place every 14 days, from 6 calls in 3 months [15] to 23 calls in 12 months [11]. In four of the five studies, subjective burden was investigated as the outcome. The same was found for depressiveness. An improvement in depressive symptoms was found in three of the four studies for the intervention group [13–15]. The intervention led to a reduction in subjective burden in only two of the four studies in which subjective burden was measured [11, 12]. Overall, there was no association between the length of the intervention phase and the result, i.e., the results of studies with longer intervention phases ranging from 5 to 12 months [11, 13, 14] were not superior to those of the studies with an intervention phase lasting only 3 months [12, 15].

The aim of this paper was to test a research hypothesis that proposed that a brief telephone intervention for informal caregivers would lead to statistically significantly greater reductions in burden and depressiveness in informal caregivers than in the control group at the end of the six-month intervention phase.

## Methods

### Study design

The DeTaMAKS study (dementia – day-care – MAKS therapy; ISRCTN16412551) was conducted as a two-arm, cluster-randomised, controlled, multicentre, prospective longitudinal study with a wait-list control group design. In this paper, we examined the effect of a brief telephone intervention for caregivers on burden and depressiveness. The study began in October 2014 and ended in March 2017. For the details of the study design (including sample size estimation, recruitment and screening process, inclusion criteria, interventions, measures, and data quality management), please see the published study protocol [8], which adhered to the SPIRIT guidelines.

During the six-month intervention phase, the caregivers in the intervention group received a brief telephone intervention by counsellors with training in psychotherapy, while the PCIs received the non-pharmacological MAKS therapy at the day-care centres [8, 16]. Caregivers in the control group did not receive any project-specific intervention. Participants of both groups were free to take part in any additional support that was offered by the German Health Care System. All procedures were examined and approved by the Ethics Committee of the Medical Faculty of Friedrich-Alexander-University Erlangen-Nuremberg (Ref. 170_14 B).

Thirty-four participating day-care centres in Germany were stratified by study region and randomly assigned to the intervention or control group at baseline by the drawing of lots.

All users were screened to determine their suitability for the project. The main inclusion criterion for the PCIs was cognitive impairment with mild to moderate dementia (Mini-Mental State Examination (MMSE) between 10 and 23) or Mild Cognitive Impairment (MMSE > 23 and a Montreal Cognitive Assessment (MoCA) score ≤ 22 [17]). Day-care users who were completely blind or deaf, suffering from cognitive decline due to diseases other than dementia (e.g. severe depression or schizophrenia), had concrete plans for institutionalisation, or were attending the day-care centre less than once a week were excluded. Another main inclusion criterion was the existence and participation of an informal caregiver. The caregiver did not need to be related to the PCI but had to provide home care without payment. If there were several caregivers (not one main caregiver), a caregiver who had not yet retired at the time was asked to take part in the study. All participating caregivers and PCIs gave their written informed consent and were free to leave the study at any time. All participants were assessed once at baseline and again at the end of the intervention period (after six months).

### Sample

**Fig. 1:**
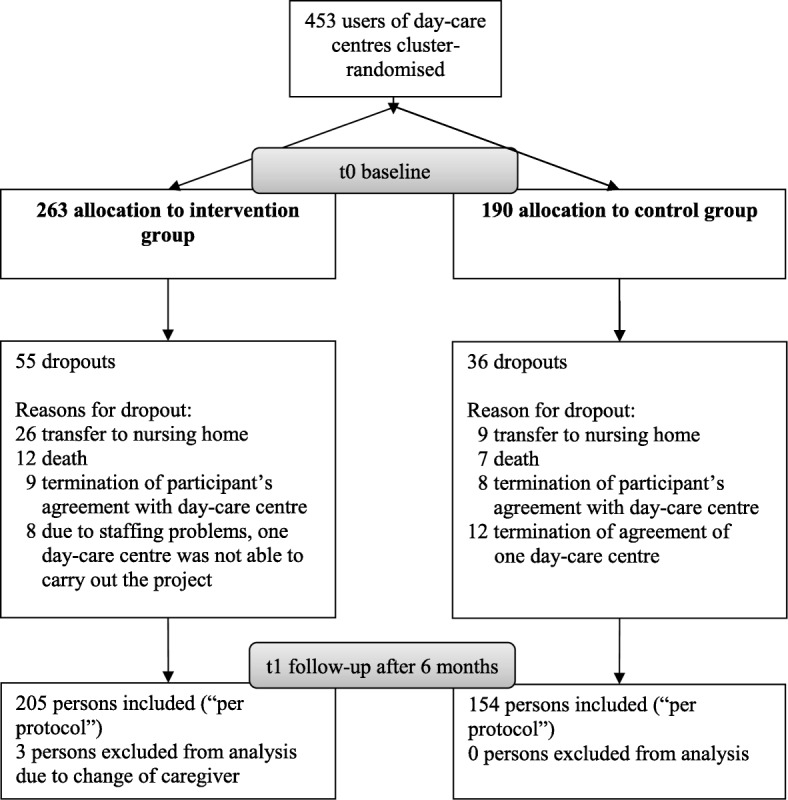
Consort flow chart

Of the 1260 screened caregiver-PCI dyads, 453 (36.0%) fulfilled our inclusion criteria and were accepted into the study. Allocation to the control or intervention group depended on the results of the cluster-randomisation. A total of 359 (79.2%) of 453 dyads completed the six-month intervention period and were included in the per protocol analysis. Their reasons for dropping out are presented in Fig. 1. The main reason was institutionalisation (38.5%). Three people had to be excluded from the analysis due to a change in caregiver. The sample for analysis consisted of two groups: 205 dyads from the intervention group and 154 from the control group.

Group analyses between caregivers of the group of 94 dropout dyads (D) and caregivers of dyads who completed the six-month intervention phase (C, *n* = 359) showed that the PCIs in the dropout subgroup were significantly older (C: mean = 81.33; SD = 7.5; D: mean = 83.13; SD = 8.2; *p* = .043). We also found higher depressiveness in caregivers of individuals in the dropout group (C: mean = 11.95; SD = 6.0; D: mean = 10.54; SD = 6.2; *p* = .043). No other significant differences were found regarding the baseline characteristics.

See Table 1 for a detailed description of the sample.

**Table 1:** Characteristics of participants (randomised at baseline, *n* = 359)

Characteristics	Intervention group	Control group	Total	Test for group differences		
	(*n* = 205)	(*n* = 154)	(*n* = 359)	*χ* ^*2*^	*T/U*	*p*
Caregiver (CG)						
Age, *M* (*SD*)	59.5 (11.4)	59.3 (11.2)	59.5 (11.3)		−0.17^a^	.868
Women, no. (%)	151 (73.7)	118 (76.6)	269 (74.9)	0.41		.521
Educational attainment (yrs.), *M* (*SD*)^b^	10.8 (2.9)	10.8 (2.8)	10.8 (2.8)		−0.23	.815
Occupation: Employed, no. (%)	111(54.1)	83 (53.9)	194 (54.0)	0.002		.962
Marital status, no. (%)				2.67		.263
Married/long-term relationship	157 (76.6)	125 (81.2)	282 (78.6)			
Widowed/divorced	26 (12.7)	20 (13.0)	46 (12.8)			
Single	22 (10.7)	9 (5.8)	31 (8.6)			
Relationship to PCI, no. (%)				1.00		.606
spouse	52 (25.4)	43 (27.9)	95 (26.5)			
son/daughter (in-law)	139 (67.8)	104 (67.5)	243 (67.7)			
other	14 (6.8)	7 (4.5)	21 (5.8)			
Caregiver burden (BSFC-s), *M* (*SD*)	11.9 (8.2)	13.2 (7.6)	12.4 (8.0)		1.55	.122
Depressiveness (WHO-5), *M* (*SD*)	12.0 (6.2)	12.0 (5.6)	12.0 (6.0)		0.02	.981
Benefits (BIZA-D), *M* (*SD*)	12.6 (4.8)	12.6 (5.4)	12.6 (5.0)		−0.13	.898
Health-related quality of life (EQ-5D-5 L),*M* (*SD*)	0.86 (0.2)	0.82 (0.2)	0.84 (0.2)		−1.67	.095
Person with cognitive impairment (PCI)						
Age, *M* (*SD*)	81.5 (7.5)	81.1 (7.5)	81.3 (7.5)		−0.61	.545
Women, no. (%)	126 (61.5)	94 (61.0)	220 (61.3)	0.01		.935
Educational attainment (yrs.), *M* (*SD*)	9.8 (2.5)	9.6 (2.1)	9.7 (2.3)		−0.56	.574
Cognitive impairment (MMSE), *M* (*SD*)^c^	19.7 (4.8)	19.3 (4.8)	19.5 (4.8)		−0.92	.357
mild cognitive impairment	25.8 (1.5)	26.3 (1.4)	26.0 (1.5)		1.27	.207
mild dementia	20.6 (1.7)	20.5 (1.6)	20.6 (1.6)		−0.31	.757
moderate dementia	14.4 (2.4)	14.6 (2.2)	14.5 (2.3)		0.28	.778
Activities of daily living (ETAM), *M* (*SD*)	17.8 (7.0)	17.1 (7.5)	17.5 (7.2)		−0.89	.373
Social behaviour (NOSGER), *M (SD)*	15.5 (4.2)	15.6 (4.6)	15.5 (4.3)		0.11	.912
Neuropsychiatric symptomatology (NPI), *M (SD)*	5.2 (2.69)	5.4 (2.7)	5.3 (2.7)		0.60	.549
Care level, no. (%)^d^					−0.19^e^	.852
None	8 (3.9)	12 (7.8)	20 (5.6)			
0	25 (12.2)	15 (9.7)	40 (11.1)			
1	109 (53.2)	75 (48.7)	184 (51.3)			
2	61 (29.8)	50 (32.5)	111 (30.9)			
3	2 (1.0)	2 (1.3)	4 (1.1)			
Use of antidementia med., no. (%)^f^	61 (29.8)	40 (26.0)	101 (28.1)	0.62		.430
Care situation						
Main caregiver, no. (%)	178 (86.8)	139 (90.3)	317 (88.3)	1.002		.317
Sole informal caregiver, no. (%)	110 (53.7)	83 (53.9)	193 (53.8)	0.002		.964
Living together, no. (%)	86 (42.0)	68 (44.2)	154 (42.9)	0.174		.676
Duration of informal care (mo.), *M (SD)*	60.3 (49.1)	59.0 (52.2)	59.7(50.4)		−0.24	.814
Frequency day-care use, *M (SD)*^*g*^	2.0 (1.2)	1.9 (1.1)	1.9 (1.2)		−0.17	.866
Informal care time per day (h), *M (SD)*^*h*^	3.0 (2.2)	3.3 (2.0)	3.1 (2.1)		1.09	.277
No. of additional Formal Care Support, *M (SD)*^*i*^	1.6 (1.3)	1.6 (1.3)	1.6 (1.3)		0.26	.799

*M* mean, *SD* standard deviation, *BIZA-D* Berlin Inventory of caregiver burden - dementia patients (score) - subscale Benefits, *BSFC-s* Burden Scale for Family Caregivers short (score), *EQ-5D-5 L* EuroQol five dimensions questionnaire, *ETAM* Erlangen test of activities of daily living in persons with mild dementia or mild cognitive impairment (score), *MMSE* Mini-Mental Status Examination (score), *NOSGER* Nurses’ Observation Scale for Geriatric Patients, *NPI* Neuropsychiatric Inventory Questionnaire *WHO-5* Well-Being Index score

^a^t-tests are reported as metric variables, u-tests were also computed but not reported because they failed to indicate a different level of significance

^b^min. 7 yrs. (no compulsory school leaving certificate) - max. 18 yrs. (university degree)

^c^mild cognitive impairment: MMSE 30–24 & Montreal Cognitive Assessment (MoCA) 0–22, mild dementia: MMSE 23–18, moderate dementia: MMSE 17–10

^d^the extent to which nursing care was needed according to the health insurance: none (no needs), 1 (moderate needs), 2 (high needs), and 3 (very high needs)

^e^u-test

^f^intake of memantine or acetylcholinesterase inhibitors

^g^average frequency per week in the first month

^h^hours of average informal care per day adjusted for day care attendance at baseline

^i^sum of formal care, support, in addition to day-care centre, maximum: 9 (caregiver skill training, counselling service for caregivers, support group for caregivers, domestic care service, care group, meals on wheels, respite care, outpatient care service, home-help service)

### Interventions

#### Brief telephone intervention for informal caregivers

In the six-month intervention phase, the caregivers from the intervention group received three outreach telephone calls that were based on a manual designed specifically for the study. The manual-guided phone calls, each lasting up to one hour, were performed at the beginning of the six-month period, after about two months, and towards the end of the intervention phase. The task of the counsellors, who had been training in psychotherapy and had received prior training specifically for the intervention, was to support the caregivers in developing strategies for self-management [18], to help reduce the stress [19] involved in providing home care, and to help the caregivers deal with challenging behaviours [20, 21]. Tried and tested procedures from stress psychology were adjusted to fit the caregivers’ situations. The aim of the intervention was to “empower” the caregivers by improving their skills. The counsellors’ basic attitude was client-centred [22] and solution-oriented. The first call was used to explore the most stressful problems and to deduce goals. With the help of problem-solving approaches, the focus was to try to improve the problems encountered in the caregiving situation in the first two calls. The third call was used as a booster session: The caregiver evaluated the phone calls, and an individual emergency tool kit was created for the future. For more information about the telephone-based caregiver intervention and a detailed description of the procedure, see the published study protocol [8] and the additional material (see Additional file 1).

During the intervention phase, 87.3% (*n* = 179) of the caregivers in the intervention group received 3 or more telephone calls (4 cases with 4 calls), 3 caregivers (1.5%) declined all telephone counselling, 2 caregivers (1.0%) were content with one telephone call, and 21 people (10.2%) felt comfortable with two telephone intervention sessions.

#### MAKS therapy for persons with cognitive impairment

In addition to the caregiver intervention, all PCIs in the intervention group received the non-pharmacological, multicomponent MAKS therapy provided by the day-care centres for 6 months. The components consisted of *m*otor stimulation, *a*ctivities of daily living, and *c*ognitive stimulation in a *s*ocial setting [16, 23, 24]. The MAKS therapy was administered Monday to Friday in groups of 10 individuals by two trained day-care centre staff members. The treatment dose of the PCIs in the intervention group was between 1 and 5 treatment days per week, depending on their contractually fixed attendance at the day-care centre, which was also not influenced by the study centre. For more information, see our study protocol [8].

### Outcomes and assessments

All data were recorded at baseline and at the end of the intervention phase after 6 months by means of CATIs [25], which were administered to the caregivers by trained interviewers (psychology students). PCI-related data that were used to assess the severity of dementia symptoms were collected by means of psychometric tests. Trained staff members who were not involved in the care of the day-care-centre users administered the psychometric tests at the day-care centres (quasiblinded). Additional PCI data (e.g. age, sex, care level) were obtained from the day-care centres’ documentation. The data quality was guaranteed by stringent data monitoring at the study headquarters for the entire period of data collection. Details of the measures employed, data collection, and data quality management are presented in the published study protocol [8].

#### Primary outcomes

The interviewer used the Burden Scale for Family Caregivers short (BSFC-s) [26] to assess informal caregivers’ subjective burden (higher values indicating greater burden) and the WHO-5 Well-Being Index (WHO-5) [27, 28], which measures well-being in terms of level of depressiveness. It is recommended as a screening tool for unipolar depression [29] and evaluates a person’s mood during the last 14 days (lower values indicating greater depressiveness).

#### Secondary outcomes

In addition, the “Benefits” subscale of the Berlin Inventory of caregiver burden with dementia patients (Berliner Inventar zur Angehörigenbelastung – Demenz, BIZA-D) [30] was used to assess positive aspects of caregiving. To obtain information about caregivers’ health-related quality of life (HRQL), the EuroQol five dimensions questionnaire (EQ-5D-5 L) [31] was used.

Utility scores were calculated by using the crosswalk value set for German time trade-off scoring algorithm created by the EuroQol Group for use until national EQ-5D-5 L value sets are available [32].

#### Other measures

The Resource Utilization in Dementia (RUD) questionnaire [33] was employed to evaluate formal and informal care, specifically the use of resources by both the PCIs and their informal caregivers. Information on family status, level of education, and the duration of the care situation were collected by means of the CATI. The screening questions of the Neuropsychiatric Inventory Questionnaire (NPI-Q) were employed to document the informal caregivers’ evaluation of the PCIs’ neuropsychiatric symptoms [34].

It can be assumed that both depressiveness and subjective burden also depend on factors other than the intervention. At t1 (after six months), major events in the caregivers’ lives during the last six months were therefore recorded (“Have there been any major events in your life/ care situation during the last six months?”) with the response options “yes” or “no”, and caregivers were asked to name the concrete event. According to Holmes and Rahe’s [35] concept, critical life events, regardless of their specific quality (e.g. positive or negative) can challenge organisms because of their adaptive demand.

Caregivers in the intervention group were also asked to evaluate the telephone intervention by rating four statements (“Through counselling, I can deal better with behaviours of the person in my care that challenge me”; “Through counselling, I have learned how to better take care of my needs”; “In the conversation, the topics that were important to me were dealt with”; “Through counselling, I have undertaken something specific to change my situation”). These intervention-related questions were rated on a scale with the anchors “strongly agree”, “neither agree nor disagree”, and “strongly disagree”.

### Statistical analyses

Descriptive methods were used to describe the sample. To assess the quality of the randomisation, non-parametric tests (U-Test, χ^2^-Test) and parametric tests (t-Test) were used to examine significant differences between the intervention and control groups at baseline [36, 37]. As the primary method of analysis, we used the per-protocol analysis (PP) to test the effect of the intervention on primary outcomes. Otherwise, the effect of the intervention would be distorted because the institutionalisation or death of the PCI would be expected to have a substantial effect on caregiver burden and depressiveness. But as a secondary analysis, we added an intention to treat analysis (ITT). Missing values in the WHO-5 and BSFC-s data from dropouts were imputed by carrying the last value forward. For the ITT analysis, the same potential confounding variables were included. We added PCIs’ age because we found that there was a significant difference in age between dropouts and cases at baseline.

Cohen’s d was calculated for all outcomes as a measure of effect size [38]. The level of statistical significance was set at *p* = 0.05. All analyses were computed with the “IBM SPSS Statistics 21” software.

#### Main analysis

To analyse the primary outcomes, we calculated multiple linear regressions, with the scores on the BSFC-s and WHO-5 after six months (t1) as dependent variables and the corresponding baseline scores for the primary outcomes and group membership as independent variables. We also included caregiver age and sex, sole responsibility for informal care (sole caregiver yes/no), and the potential confounding variables. These potential confounders were the frequency of day-care use per week during the six-month intervention phase, the total number of other counselling services or relief services used, and the change in PCIs’ neuropsychiatric symptoms between baseline and the 6-month measurements. All variables fulfilled the criterion of no multicollinearity.

Participants were identified as persons with favourable change if there was a reduction or as persons with unfavourable change if there was no change or an increase in subjective burden and depressiveness. For subjective burden, a favourable change was defined as having a positive difference between the BSFC-s baseline score (t0) and the score after 6 months (t1) and an unfavourable change as showing a negative difference or an unchanged BSFC-s score. For depressiveness, responders had a negative difference between t0 and t1 in their WHO-5 score, whereas non-responders showed unchanged or positive differences. We compared the distributions of responders and non-responders for both outcomes in the control and intervention groups using χ^2^-Tests.

#### Sensitivity analysis

As a sensitivity analysis, we applied the same multivariate analysis strategy to cases that were not affected by events that caregivers subjectively identified as major events during the six-month intervention phase.

#### Subgroup analysis

An exploratory subgroup analysis was also computed for these cases. The three subgroups were caregivers of people with MCI or mild or moderate dementia. We therefore computed change scores (the differences in the means of the primary outcomes between t0 and t1) and compared the scores with *t*-tests for independent samples.

#### Analysis of secondary and other outcomes

In the same way as described above, change scores were computed for the secondary outcomes as the differences between t0 and t1 and compared with the aid of a *t*-test for independent samples. Data on intervention-related questions were analysed with descriptive methods.

## Results

No significant differences between the control and intervention groups (*n* = 359) were found for any variable at baseline (see Table 1).

### Primary study outcomes: Effects of the DeTaMAKS telephone counselling on caregiver burden and depressiveness

The means of the BSFC-s (mean = 12.4; SD = 8.0) and WHO-5 (mean = 12.0; SD = 6.0) scores at baseline were in the middle of the respective ranges (BSFC-s: 0–30; WHO-5: 0–25). The probability of a tendency towards a floor or ceiling effect could therefore be considered low. In the analysed sample (*n* = 359), no significant effect of the brief telephone intervention was found for the two main dependent variables in the multiple linear regression analysis (BSFC-s *p* = .128, WHO-5 *p* = .107; Table 2). The respective baseline scores of the primary outcomes were the only significant predictors of the outcomes after six months. Descriptively, more caregivers in the intervention group (I) than in the control group (C) had a more favourable change in subjective burden rather than an unchanged or increased burden over the course of the six-month intervention period (reduction BSFC-s: I: 51.7% vs. C: 41.6%, χ2 = 3.63, *p* = .057; WHO-5: I: 48.8% vs. C: 44.8%, χ2 = 0.56, *p* = .455). In the ITT analysis, there were also no statistically significant values for the variable “group membership” for both outcomes (*p* = .097 for BSFC-s; *p* = .108 for WHO-5).

**Table 2:** Multiple regression analysis with BSFC-s and WHO-5 scores after 6 months (t1) as dependent variables (*n* = 359)

Independent variable	BSFC-s (6-month follow up)				WHO-5 (6-month follow up)			
	Unstand. *b*	*p*	95% CI		Unstand. *b*	*p*	95% CI	
			lower	upper limit			lower	upper limit
Score at baseline^a^	0.84	.000*	0.77	0.90	0.60	.000*	0.51	0.68
Group (0 = control group, 1 = intervention group)	−0.74	.128	−1.69	0.21	0.83	.107	−0.18	1.83
Age of caregiver	−0.00	.866	−0.05	0.04	−0.03	.250	−0.08	0.02
Sex of caregiver (0 = female; 1 = male)	−0.27	.631	−1.35	0.82	−0.21	.724	−1.36	0.95
Frequency of day-care use^b^	−0.09	.689	−0.53	0.35	0.12	.607	−0.35	0.59
Other relief services^c^	0.06	.785	−0.36	0.48	−0.02	.926	−0.47	0.43
Other counselling services^d^	0.14	.801	−0.93	1.20	−0.46	.419	−1.57	0.65
Sole informal caregiver (0 = no, 1 = yes)	−0.99	.067	−2.04	0.07	−0.30	.598	−1.40	0.81
Change in PCIs’ neuropsychiatric symptoms^e^	−0.14	.915	−0.27	0,24	−0.21	.127	−0.48	0.06

Significant *p*-values (<.05) are shown in bold and marked with *

*Abbreviations*: *BSFC-s* Burden Scale for Family Caregivers, short version (score) *WHO-5* Well-Being Index score *PCI* person with cognitive impairment

^a^BSFC-s at baseline if BSFC-s 6-month follow-up is dependent variable, WHO-5 at baseline if WHO-5 6-month follow-up is dependent variable

^b^average frequency per week (month 1–6)

^c^sum of domestic care service, care group, meals on wheels, respite care, outpatient care service, home-help service

^d^sum of caregiver skill training, counselling service for caregivers, support group for caregivers

^e^computed via Neuropsychiatric Inventory Questionnaire (NPI-Q), change score as the difference between the NPI score at baseline and after 6 months, positive values on the NPI change score indicate improvements in neuropsychiatric symptoms

### Sensitivity analysis

Eighty-eight (24.5%) of the caregivers in the analysed sample (C: 21.4% vs. I: 26.8%; χ2 = 1.39, *p* = .239) reported that they had experienced at least one major event. The three most frequently mentioned events were: illness/accident experienced by family members/friends (*n* = 25, 28.4%), illness/accident experienced by the caregiver (*n* = 12, 13.6%), or a marked deterioration in the PCIs’ state of health (*n* = 9, 10.2%). No significant differences between the control and intervention groups (*n* = 271) were found for any variable at baseline for this subgroup despite the baseline value of EQ-5D (I: mean = 0.87; SD = 0.2; C: mean = 0.82; SD = 0.2; *p* = 0.015). A comparison of the WHO-5 and BSFC-s baseline scores between the subgroups of people with major events (M; *n* = 88) and no major events (N; n = 271) during the intervention period showed no difference in depressiveness at baseline (M: mean = 11.3; SD = 5.3; N: mean = 12.15; SD = 6.1; *p* = .268), but there was a difference in caregiver burden (M: mean = 13.99; SD = 7.6; N: mean = 11.93; SD = 8.0; *p* = .035). Caregivers who experienced a major event during the 6-month intervention phase had significantly higher burden scores at baseline than caregivers who did not experience a major event.

The multivariate regression analyses for the 271 informal caregivers who did not experience a major event showed that the brief telephone intervention was a significant predictor of improvement in both subjective burden and depressiveness six months later (BSFC-s adjusted mean difference: -1.3, 95% CI -2.4 to − 0.3, *p* = .010, Cohen’s d = 0.27; WHO-5: adjusted mean difference: 1.54, 95% CI 0.4 to 2.7, *p* = .008, Cohen’s *d* = 0.26; Table 3). Apart from the intervention, only the values of the outcome variables at baseline were significant predictors of the respective values after six months. None of the following were significant predictors: age, sex, frequency of day-care use, use of other relief or counselling services, sole responsibility for caregiving (yes/no), or changes in PCIs’ neuropsychiatric symptoms. The null hypothesis could therefore be accepted for the total sample (*n* = 359) but not for the subsample (*n* = 271). For this subsample, it could be concluded that the brief telephone intervention had a significantly favourable influence on both the subjective burden and the depressiveness of the caregivers (see Tables 2 and 3).

**Table 3:** Multiple regression analysis with BSFC-s and WHO-5 after 6 months (t1) as dependent variables for cases without major events (*n* = 271)

Independent variable	BSFC-s (6-month follow up)				WHO-5 (6-month follow up)			
	Unstand. *b*	*p*	95% CI		Unstand. *b*	*p*	95% CI	
			lower	upper limit			lower	upper limit
Score at baseline^a^	0.89	.000*	0.82	0.96	0.55	.000*	0.45	0.64
Group (0 = control group, 1 = intervention group)	−1.34	.010*	−2.35	−0.33	1.54	.008*	0.41	2.67
Age of caregiver	−0.02	.483	−0.07	0.03	−0.05	.098*	−0.10	0.01
Sex of caregiver (0 = female; 1 = male)	−0.63	.280	−1.78	0.52	0.20	.764	−1.09	1.49
Frequency of day-care use^b^	0.20	.400	−0.27	0.68	0.12	.651	−0.41	0.65
Other relief services^c^	−0.14	.557	−0.60	0.33	0.11	.675	−0.41	0.63
Other counselling services^d^	−0.19	.758	− 1.38	1.01	−0.58	.378	−1.89	0.72
Sole informal caregiver (0 = no, 1 = yes)	− 0.67	.252	−1.81	0.48	−0.30	.638	−1.56	0.96
Change in PCIs’ neuropsychiatric symptoms^e^	0.03	.856	−0.24	0.29	−0.29	.059	−0.59	0.01

Significant *p*-values (<.05) are shown in bold and marked with *, *p*-values below .1 are marked with *

*Abbreviations*: *BSFC-s* Burden Scale for Family Caregivers short (score) *WHO-5* Well-Being Index score *PCI* person with cognitive impairment

^a^BSFC-s at baseline if BSFC-s 6-month follow-up is dependent variable, WHO-5 at baseline if WHO-5 6-month follow-up is dependent variable

^b^average frequency per week (months 1–6)

^c^sum of domestic care service, care group, meals on wheels, respite care, outpatient care service, home-help service

^d^sum of caregiver skill training, counselling service for caregivers, support group for caregivers

^e^computed via Neuropsychiatric Inventory Questionnaire (NPI-Q), change score as the difference between the NPI score at baseline and after 6 months, positive values on the NPI change score indicate improvements in neuropsychiatric symptoms

### Subgroup analysis

**Fig. 2:**
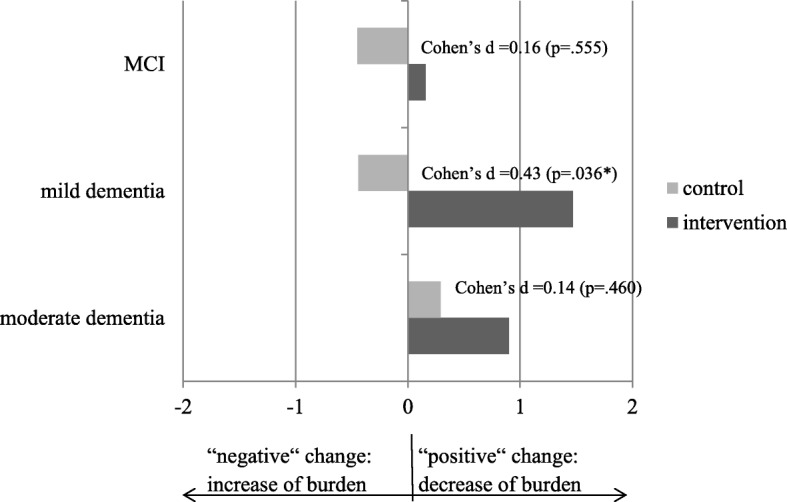
Pre-post differences in caregiver burden (BSFC-s), for three categories of cognitive impairment (*n* = 271). Cases with a major event during the 6-month intervention phase were excluded. Change scores were computed (the differences in the means of the primary outcomes between t0 and t1). Positive values indicate a decrease in caregiver burden (e.g. + 1 means on average one point less on the burden score after the 6-month intervention phase). MCI (*n* = 54): Intervention M = 0.16, SD = 3.84, Control M = − 0.45, SD = 3.52. Mild Dementia (*n* = 110): Intervention M = 1.47, SD = 3.32, Control M = − 0.44, SD = 5.53. Moderate Dementia (*n* = 107): Intervention M = 0.90, SD = 4.19, Control M = 0.29, SD = 4.32. Significant *p*-values (<.05) are marked with *

**Fig. 3:**
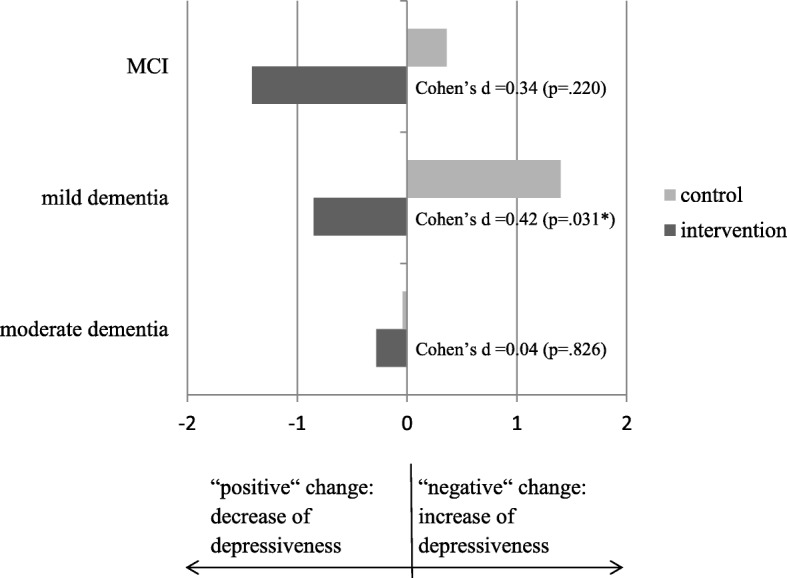
Pre-post difference in depressiveness (WHO-5), for three categories of cognitive impairment (*n* = 271). Cases with a major event during the 6-month intervention phase were excluded. Change scores were computed (the differences in the means of the primary outcomes between t0 and t1). Negative values indicate improvements in symptoms (e.g. -1 means on average one point more on the WHO-5 Well-Being Index after the 6-month intervention phase). MCI (*n* = 54): Intervention *M* = − 1.41, SD = 5.71, Control *M* = 0.36, SD = 4.18. Mild Dementia (*n* = 110): Intervention *M* = − 0.85, SD = 4.88, Control *M* = 1.40, SD = 5.92. Moderate Dementia (*n* = 107): Intervention *M* = −.28, SD = 4.86, Control *M* = − 0.04, SD = 6.16. Significant *p*-values (<.05) are marked with *

A comparison of the changes in the burden on caregivers (BSFC-s) and their depressiveness (WHO-5) in the intervention group with those in the control group when taking the severity of the cognitive impairment into consideration showed significant differences for mild dementia (BSFC-s: Cohen’s d = 0.43, *p* = .036; WHO-5: Cohen’s d = 0.42, *p* = .031; Figs. 2 and 3). The effect of the brief telephone intervention on the caregiver outcomes was thus greatest in this range of severity (see Figs. 2 and 3).

After six months, the brief telephone intervention was assessed by the caregivers in the intervention group who had received at least one phone call (*n* = 147) in response to four intervention-related questions on the CATI (see Table 4).

**Table 4:** Intervention-related outcomes: evaluation of the brief telephone intervention (*n* = 147)

Changes due to brief telephone intervention	Scale	Severity of cognitive decline			Total
		MCI^a^	Mild dementia^b^	Moderate dementia^c^	
		(*n* = 31)	(*n* = 60)	(*n* = 56)	(*n* = 147)
Challenging behaviours^d^, no.(%)	Strongly agree	14 (45.2)	31 (51.7)	20 (35.7)	65 (44.2)
	Neither agree nor disagree	11 (35.5)	18 (30.0)	14 (25.0)	43 (29.3)
	Strongly disagree	6 (19.4)	11 (18.3)	22 (39.3)	39 (26.5)
Own needs^e^, no.(%)	Strongly agree	15 (48.4)	27 (45.0)	19 (33.9)	61 (41.5)
	Neither agree nor disagree	10 (32.3)	23 (38.3)	20 (35.7)	53 (36.1)
	Strongly disagree	6 (19.4)	10 (16.7)	17 (30.4)	33 (22.4)
Dealing with important Topics^f^, no.(%)	Strongly agree	21 (67.7)	52 (86.7)	35 (62.5)	108 (73.5)
	Neither agree nor disagree	6 (19.4)	6 (10.0)	11 (19.6)	23 (15.6)
	Strongly disagree	4 (12.9)	2 (3.3)	10 (17.9)	16 (10.9)
Specific changes in care situation^g^, no.(%)	Strongly agree	10 (32.3)	19 (31.7)	21 (37.5)	50 (34.0)
	Neither agree nor disagree	5 (16.1)	7 (11.7)	8 (14.3)	20 (13.6)
	Strongly disagree	16 (51.6)	34 (56.7)	27 (48.2)	77 (52.4)

Cases from the intervention group with no subjective outstanding event during the 6-month intervention phase were included in the analysis 3 cases were excluded because the caregiver declined the caregiver intervention

^a^MCI: baseline score on the Mini-Mental State Examination (MMSE 30–24) & Montreal Cognitive Assessment (MoCA) 0–22

^b^Mild dementia: baseline score on the MMSE 23–18

^c^Moderate dementia: baseline score on the MMSE 17–10

^d^“Through counselling, I can deal better with behaviours of the person in my care that challenge me”

^e^“Through counselling, I have learned how I can better take care of my needs”

^f^“In the conversation, the topics that were important to me were dealt with”

^g^“Through counselling, I have undertaken something specific to change my situation”

Caregivers of people with MCI or mild dementia more frequently agreed that the telephone intervention had helped them to cope better with the PCIs’ challenging behaviours. The same applied to the question about whether the intervention had helped them to learn to take better care of their own needs. The caregiver counsellors were able to address and work on subjectively important subjects more often in the group of caregivers of people with mild dementia than in the group of caregivers of people with MCI or moderate dementia. In all three subgroups, roughly one third of the caregivers either completely or partly agreed that they had made concrete changes in their situations (see Table 4).

The patterns of responses to Questions 1 to 3 thus corresponded roughly to the effects (Cohen’s d) of the brief telephone intervention on the caregivers’ subjective burden and sense of depressiveness (see Figs. 2 and 3), i.e. the group of caregivers of people with MCI, and in particular, the caregivers of people with mild dementia assessed the brief telephone intervention more positively than the caregivers of people with moderate dementia.

### Secondary study outcomes: Effects of DeTaMAKS on caregivers’ health-related quality of life (HRQL) and benefits

In the total sample (*n* = 359), for the two secondary dependent variables (i.e. HRQL and benefits), no significant differences were found with regard to the differences between t0 and t1 in either the intervention or the control group (t-test for independent samples: HRQL: *p* = .105; benefits: *p* = .953; see Table 5).

**Table 5:** Secondary outcomes (*n* = 359)

Scale	Intervention group (*n* = 205)	Control group (*n* = 154)	*t*-test for independent samples	
	*∆M* (*SD*)	*∆M* (*SD*)	*t*	*p*
Health-related quality of life (EQ-5D-5 L)	.00 (0.2)	−.03 (0.2)	t(357) = −1.625	.105
Benefits (BIZA-D)	−.29 (4.0)	−.27 (4.5)	*t*(357) = .059	.953

Comparison of differences between baseline and after 6 months. Negative values on the BIZA-D change score (t0-t1) indicate improvements in seeing positive aspects of caregiving, negative values on the EQ-5D-5 L change score (t0-t1) indicate improvements in health-related quality of life *p-values < .05

## Discussion

In the DeTaMAKS project, a brief telephone intervention for informal caregivers was developed and evaluated. The intervention showed a statistically significant reduction in depressiveness and burden in the subgroup of caregivers who had not experienced any major life events (according to Holmes and Rahe [35]; e.g. own illness/accident, a general deterioration in the state of health of the person requiring care) during the six-month intervention period. By contrast, for the total sample, the respective baseline value of the primary outcome was the sole significant predictor. The ITT analyses, which included dropouts, also showed no significant values for group allocation in either outcome.

The strongest effect of telephone counselling for informal caregivers in the “low-dose” form carried out in our study was observed in the early stages, that is, in mild cognitive impairment and particularly in mild dementia, whereas it was much less marked in caregivers who were caring for a person with moderate dementia at home. The evaluation of the telephone counselling for caregivers by means of intervention-related questions supported this finding because informal caregivers of people with MCI or mild dementia more often reported that the telephone counselling helped them to cope better with PCIs’ challenging behaviours and to pay more attention to their own needs.

The brief telephone intervention is a low-dose intervention. To have an effect on caregiver burden and the depressiveness of people with moderate dementia, we suggest that a higher dose of a telephone intervention is needed. For caregivers of people with MCI to mild dementia, a higher dose is also needed to help them overcome the effect of events that were experienced as major events.

Also the comparison with other studies reported in the literature suggests that, for informal caregivers of people with moderate dementia, an intervention with a broader spectrum and a higher “dose” of counselling is required to achieve significant effects.

Providing caregivers with telephone support offers some advantages in the day-care setting. Telephone support can be provided to caregivers at little expense and with a very low threshold, it is an outreach intervention and does not require the caregivers to show up somewhere in person, and it is provided by qualified counsellors without time pressure and with a high level of flexibility with respect to time of day. Because it is an outreach programme, and thus, there is also the chance that it may achieve a preventive effect [39]. Moreover, the telephone setting offers a protective frame in which the caregivers have control over whether they will take advantage of the service.

It is interesting that none of the comparison studies from our literature search reported that they had carried out an analysis of the responders. We found that when caregivers reported experiencing major events in their lives and caregiving situations, these events were “superimposed” on the effect of an intervention on depressiveness and stress. Caregivers who experienced a major event during the 6-month intervention phase had significantly higher burden scores at baseline than caregivers who did not experience a major event. Some major events could have been triggered by this higher burden score (e.g. more physical psychosomatic complaints, see Pendergrass et al. [40]). But the higher score cannot completely account for the experience of a major event, because we could not find a difference in depressiveness. Thus, this finding is a novel insight. Moreover, the treatment effect in our study was manifested in the subgroup without major events at a much lower “treatment dose” (i.e. three phone calls in six months) than in the studies cited above. Our results also show that it is necessary to consider different courses of outcomes in subgroups of caregivers.

In the same period in which the telephone counselling with the caregiver was carried out, the individuals with cognitive impairment received the multi-component, non-drug MAKS therapy at the day-care centres. Straubmeier et al. [41] found that the PCIs’ cognitive skills and abilities to carry out activities of daily living were stabilised during the 6-month intervention period. The neuropsychiatric symptoms (existing or not existing) of the PCIs showed a more favourable development in the intervention group.

There is currently little evidence that would help in deciding whether an intervention for PCIs has a significant effect on depressiveness and burden in their informal caregivers. In an ongoing randomised controlled study in which individuals with early-stage dementia underwent cognitive rehabilitation in 14 therapy sessions, positive effects of the intervention on the PCIs were reported. However, there were no differences between the secondary caregiver-related outcomes stress and quality of life in the control and intervention groups [42]. Another study that investigated potential indirect effects of cognitive stimulation in people with dementia on the health and health-related quality of life of their caregivers came to the same conclusion [43].

We postulate that the changes in ADL and cognition from the MAKS therapy are not noticeable for caregivers in their daily routine because it is a stabilising effect of the intervention (ETAM and MMSE scores remain constant on average). The effect of the intervention arose from the fact that ADL and cognition decreased on average in the control group over 6 months (mean decrease in MMSE: one point in 6 months).

But we assume that a significant change in PCIs’ neuropsychiatric symptoms (presence/absence of symptoms) could bring noticeable subjective relief to the caregiver.

Therefore, we added the variable “change in neuropsychiatric symptoms” to the multiple regression analysis to test the potential influence of change in PCIs’ neuropsychiatric symptoms on subjective caregiver outcomes. The results showed that PCIs’ change in neuropsychiatric symptoms was not a significant predictor of caregiver burden or depressiveness.

Thus, on the whole, the effect of the caregiver intervention cannot be attributed to the change in PCIs’ neuropsychiatric symptoms. However, for a final clarification of a potential confounding effect of the MAKS therapy on the caregiver telephone intervention, a new study with separately administered single interventions is necessary.

### Strengths and limitations

The strengths of this study are its randomised control design, the large number of participants, and a high level of external validity because existing care structures (i.e. day-care centres) were used to reach the caregivers. The brief telephone intervention we employed was in a manualised form, but it could still be individualised in the sense that it was possible to work on the challenging behaviours that the caregiver identified as relevant or the specific triggers of stress they mentioned. Various research reports have shown that this kind of individualised intervention is more effective than a “one size fits all” intervention [44, 45].

Limitations resulted from the fact that the caregiver data are based on self-report measures. They are thus subject to the usual errors of judgement, which, however, also apply equally for persons in both the intervention group and the control group. The generalisability of the results is limited insofar as it is restricted to the subgroup of caregivers who use a day-care centre for the PCI.

### Prospects for future research

In order to increase the generalisability of the results, further studies on this brief telephone intervention need to be carried out in different samples, which will, however, ensure that the caregivers can be reached as early as possible. For example, this would be the case for caregivers who use an outpatient care service. Future studies are still needed to determine what effect an issue-focussed telephone intervention for caregivers would have if it were offered over a longer period of time or were more intensive or how it would have to be structured so that it would also be effective for caregivers of individuals with moderate dementia.

## Conclusions

The results of a manualised telephone intervention for caregivers oriented towards stress reduction and the development of strategies for self-management and dealing with challenging behaviours showed significant improvements in depressiveness and subjective burden but only if major events in the caregivers’ lives were also taken into account. It is recommended that a telephone intervention begin at an early stage because the effects were much stronger when the PCIs had mild dementia rather than moderate dementia.

## Additional file

Additional file 1: Guideline: Brief telephone intervention for informal caregivers. (DOCX 47 kb)

## Abbreviations

ADL: Activities of daily living
BIZA-D: Berlin Inventory of caregivers’ burden of dementia patients
BSFC-s: Burden Scale for Family Caregivers short
CATI: Computer-assisted telephone interview
DeTaMAKS: A cluster-randomised, controlled intervention study examining MAKS therapy for persons with cognitive impairment in day-care centres
EQ-5D-5 L: EuroQol five dimensions questionnaire
ETAM: Erlangen test of activities of daily living in persons with mild dementia or mild cognitive impairment
HRQL: Health-related quality of life
MAKS: Multicomponent group therapy for persons with cognitive impairment (motor stimulation, activities of daily living and cognitive stimulation in a social setting)
MCI: Mild cognitive impairment
MMSE: Mini-Mental State Examination
MoCA: Montreal Cognitive Assessment
NOSGER: Nurses’ Observation Scale for Geriatric Patients
NPI-Q: Neuropsychiatric Inventory Questionnaire
PCI: Person with cognitive impairment
RUD: Resource Utilization in Dementia
WHO-5: Well-Being Index
