# Supplementary material for: Brief telephone counselling is effective for caregivers who do not experience any major life events – caregiver-related outcomes of the German day-care study
Source: BMC Health Serv Res. 2019 Jan 9;19:20. doi: 10.1186/s12913-018-3853-8 (PMC6325874; doi:10.1186/s12913-018-3853-8)
Supplement: Supplementary file 1 — Guideline: Brief telephone intervention for informal caregivers. (DOCX 47 kb) [file 12913_2018_3853_MOESM1_ESM.docx]

# Guideline: Brief telephone intervention for informal caregivers

# Phone Call No. 1

| Contents   - Determination of setting and establishment of a relationship   - Explanation of the background - Exploration of problem areas, the immediate environment, and potential interventions   - Problems, placing the focus on the perspective of the caregiver (own issues)   - Focus on possible solutions - End of session   - Summary of the session   - Finding an intervention-related exercise for the time between the sessions   - Setting a new appointment |
| --- |

1. First contact

- *“This is … ( name) from University Hospital Erlangen. In the next 6 months, we have time for 3 sessions to talk about your situation with your person in care.*  *Do you have time now for the first session? We would be happy to agree on a new time.”*
- An unusual situation. Caregivers usually get in touch when they need help/support and now it's exactly the opposite.
  - "*We will call you up to three times and would like to give you support in your care context where you need it or where you might require it in the future.”*
- *"What would you like to get from the phone calls? What are your expectations?"*
- Explain the 2-expert model, the caregiver is the expert for her/his situation

1. Anamnesis

- Before starting the call, obtain the preliminary sociodemographic data from the print-out of the computer-assisted telephone interview (CATI), which was conducted before the actual start of the study.
- Create a link to the CATI:
  - *"A colleague on my team has already called you and asked you some questions about your situation."*
  - *"You've been caring for your mother for x years ..."*
  - *"So you're /you are caring for / living ..."*

1. Problem analysis & goal analysis

- *“How are you finding the care situation?"*
- *"Are there areas that you are finding challenging/difficult/the most difficult at the moment?"*

| **Yes** | **No** |
| --- | --- |
| Description of the problem area or several areas   - if there are several: create a hierarchy and work on the most important - address the others in the following sessions or by referral to specialised services   If "only" one area occurs to the caregiver or the response does not provide openings:  see procedure labelled "no" for an alternative | *"Can you describe a typical day for me, one on which you have a lot to do for the person you are caring for? I am particularly inter­ested in what you experience and the thoughts that you have in the different situations.*  *Perhaps you or I will then see an area in which we can support you"*  Filter out of the daily schedule certain situations that you find stressful or difficult and give feedback. If appropriate also ask: *How well do you sleep at night? When did you last have time for yourself/your children? How can I support you?*   - Create a hierarchy - goal analysis   OR:  If everything is going "fine" with the care:   - *What is going well in the care you provide at home? Name 1 or 2 things that could be going better.* - *What tips would you give other people in a similar situation? What are the things / situations that you have managed to learn as time has gone on?* |

- When problem areas have been established specify them in relation to problem-solving approaches:
  - *"What would you need in XY situation to make it bearable?"*
  - *"Do you have any idea what you might need that would help you to handle XY situation better?"*
  - *"In which moments are you able to handle this situation a bit more easily?"*

You might be able to find **possible solutions** in the following areas:

For each of Points 1 to 3, there is a separate detailed document for further counselling

| 1. Understanding why a certain behaviour occurs  - general psychoeducation - handling challenging behaviours |
| --- |
| 1. Finding points of departure for coping with challenging behaviour  - situation analysis |
| 1. Coping with stress  - Gaining relief through understanding (plus 1&2) |

| **Principles: What must I pay attention to?**   - Collecting the problems must not become criticism! Most caregivers do too much and first need to experience being appreciated for that before they may be able to do somewhat less. - Filtering out of particularly challenging situations and stressful moments.   - What are the most stressful problems?   - Create a hierarchy and work on the areas one after the other - Explore hurdles, resources, and possible solutions   - What resources are recognisable / can be expanded upon?   - Are there phases of recuperation? When? Where are these possible? - Is there a need for information?   - Is psychoeducation needed? If so, in what area? - Ask the caregiver to describe the situation exactly and on different levels   - Thoughts, feelings, behaviour |
| --- |

1. Concluding the 1st phone call

- Find the intervention-related exercise by the next time: "*Our experience with similar phone sessions has shown us that it can do clients good to make use of the time between calls. Could you perhaps imagine* ....”
- Or: *Perhaps it would be a good idea if next time you were to report to me what has happened about .... When should I call you?”*
- Examples of exercise assignments
  - Obtain information about specific aid / services and report back in the 2nd phone call
  - Draw up a weekly plan: Type P (obligations) & F (free time) activities.
    - Caregiver's own needs and free time versus obligations.
    - How many obligations are there and how often is there free time per day? For how long?
  - Do something nice and report on it
  - What did I use the day-care centre time to do?
- Fix another appointment in 2 to 3 months (as required)

# Phone Call No. 2

- Link back to the last session and summarise
- Stabilise the relationship
- Come back to the issues and moods of the last session again
- Has the caregiver retained anything from the last session? How did s/he feel after it? Did any more open questions arise?
- How have things gone up to now? Have there been any changes?
- Was there an intervention-related exercise? If so, evaluate it.
- Cf. the process for Session 1: problem - goal analysis, conclusion

# Phone Call No. 3

- Evaluation of the previous phone calls
  - What has worked well?
  - Where are there still problems?
- Create an individual emergency tool kit ("case containing possibilities and options"; here there was a detailed separate document for further counselling)
  - What are ideas for a later date that I would like to note?
  - What future situations could challenge you? How can you respond? What would you then like to think of / what would you like to remember?
  - What do I do if ...?
  - Who can I turn to if ....?
